# On the Etiology and Clinical Features of the Pneumonia Occuring in the Hospital, Together with Notes Concerning the Place and Mode of Infection in the Case of Postmeasles and Bronchopneumonia

**Published:** 1918-11

**Authors:** 


***On the Etiology and Clinical Features of the Pneumonia Occuring in the Hospital, Together with Notes Concerning the Place and Mode of Infection in the Case of Postmeasles and Bronchopneumonia.*** By COLE and MAC CALLUM. *The Journal of the American Medical Association,* April 20, 1918.

During the year of 1918 much work was carried out in the United States on the subject of pneumonia, with special reference to its prevalence in the numerous army camps. This pneumonia was found to consist of two sorts, the first appearing chiefly during the pneumonia season, and consisting mainly of the acute lobar type; and the second appearing in the late winter and spring, usually complicating measles, and forming the large group of cases termed by Cole and Mac Callum as " interstitial broncho-pneumonia ", very often associated with empyema. Their work has been taken as a standard of reference by all other investigators on this subject, and by giving a brief resume of it, the outstanding features of the whole situation can be brought out.

Cole and Mac Callum were sent to Fort Sam Houston, at San Antonio, Texas, to investigate an outbreak of measles and pneumonia occurring at that place in the early spring. Upon their arrival they found by far the greater number of cases of pneumonia occurring there were of the so-called interstitial type. These studies were carried out from an epidemiological and clinical standpoint by Dr. Cole, and from a pathological standpoint by Dr. Mac Callum.

Dr Cole found that, aside from a certain number of cases of clear cut lobar pneumonia, always associated with one of the four groups of pneumococcus, there were a large number of cases occurring as sequelae to measles, which gave a clinical picture quite different from that of the ordinary pneumonia. From the sputum of these cases, and also from blood cultures made during life, and tissues after death, streptococcus haemolyticus was the organism most commonly isolated, although B. influenzae was present in a few cases. On further study it was found that the streptococcus haemolyticus was present in the throats of a small number of the patients on their entrance into the hospital, and on re-swabbing at intervals during their stay in the hospital, the proportion of positive carriers was found to increase considerably, which pointed strongly to a hospital infection. Studies of this nature were also carried out in the tuberculosis and pneumonia wards, with similar but not such pronounced results. It seemed that the presence of respiratory affections furthered the growth and development of the streptococcus, which of itself might or might not be pathogenic. These conditions were especially favorable in the case of measles, which produces, as is well known, a coryza, conjunctivitis and laryngitis.

From a pathological standpoint, the changes produced may be divided into five groups : (1) interstitial broncho-pneumonia, (2) lobular pneumonia, (3) lobar pneumonia, (4) empyema and (5) combined infections.

(1) This so-called interstitial broncho-pneumonia is a well known condition, especially in children, as a sequel of measles, whooping cough, scarlet fever, etc., and has been described from that standpoint by Bartels, Delafield, Steinhaus, and Hecht. It was also described by military surgeons during the war of 1812, as occurring among troops stationed in New-York State.

In the early stage the lung is air-containing, and on section small congested focci may be seen, usually containing a greyish centre. These lesions are very small, and microscopically are found to represent the ends of bronchioles, with their adjacent alveoli, these areas being filled with leucocytes and some short chained streptococci. Later these nodules become grey, with depressed or hollow centers, and closely resemble miliary tubercles, often projecting above the cut surface of the lung. Microscopically, in addition to the exudate of leucocytes, the bronchiole wall is found filled with an infiltration of the mononuclear type of cells, but very few streptococci being present. It is at this point that fibrinopurulent pleurisy occurs, in which streptococci are present in large numbers. As the process goes on, the lung becomes very much collapsed, dark and atelectatic, due mainly to the pressure of the pleural exudate, and on palpation some shot-like nodules may be felt throughout the lung. There is thickening of the interlobular septa, and the terminal bronchioles project very plainly from the surface, with some thickening of the tissues about them. At this time the exudate consists almost wholly of mononuclears, save for the lumina of the bronchioles, which still contain leucocytes and some streptococci; also at this stage, organization of the exudate may begin, and very often proceeds very rapidly.

(2) Lobular pneumonia. In those cases in which there was an overwhelming infection, the lungs presented the more usual irregular, patchy type of broncho-pneumonia, the distribution of which was not limited in size, or peribronchial, and in the exudate of which large numbers of streptococci could be demonstrated. Areas of necrosis with abscess formation sometimes occurred.

(3) Lobar pneumonia. The pathological picture in these cases was perfectly typical of acute lobar pneumonia, and in all cases was caused by the presence of the pneumococcus. This organism, unlike the streptococcus, occurred in large numbers in the exudate and in the alveoli, and could be seen undergoing active phagocytosis. Like the cases of streptococcus infection the organisms could be found in the lymph channels, the walls of the blood vessels, and the interlobular septa, which probably accounts for the infection of the pleura.

(4) Empyema. Out of the 37 cases of Cole and Mac Callum's series of interstitial broncho-pneumonia, empyema occurred in 26, in every case of which streptococcus haemolyticus was demonstrated. In these cases the exudate appeared early, and was usually a thin, turbid, greenish fluid, with shreds of fibrin floating in it, and with a thin layer of fibrin adherent to the lungs. Large amounts of fluid were present, and accumulated rapidly, even after repeated aspirations. Examinations of this fluid showed it to contain a surprisingly large number of organisms.

(5) Combined Infections. A few cases of combined infections, both of the lobar and interstitial broncho-pneumonia, were reported, but these were rather uncommon, and the writers thought that these two conditions tended to exclude one another.

Complications, except for the other serous activities, were uncommon.

*Summary.* Two groups of pneumococcic infection have been described, the one of the acute lobar type, the other of the interstitial bronchial type. Of these two the latter is very commonly complicated by the presence of empyema. The organism occurring in this type was found to be a hemolytic streptococcus, whereas in the case of the lobar pneumonia, pneumococcus of one type or another was always present.

